# Natural Phenolic Antioxidants from *Micromeria aybalae*: Solvent-Dependent Composition and Bioactivity

**DOI:** 10.3390/ijms27209241

**Published:** 2026-10-18

**Authors:** Zeyneb Karakus, Cengiz Sarikurkcu

**Affiliations:** 1 Chemical Technology Program, Çay Vocational School, Afyon Kocatepe University, 03700 Afyonkarahisar, Türkiye; 2 Department of Analytical Chemistry, Faculty of Pharmacy, Afyonkarahisar Health Sciences University, 03030 Afyonkarahisar, Türkiye

**Keywords:** *Micromeria aybalae*, rosmarinic acid, phenolic profiling, antioxidant activity, LC–MS/MS

## Abstract

*Micromeria aybalae* H.Duman & Dirmenci is a recently described endemic Lamiaceae species from southwestern Türkiye whose non-volatile phytochemistry and biological properties remain largely unexplored. This study comparatively evaluated ethyl acetate, methanol, and water extracts of its aerial parts for phenolic composition, antioxidant capacity, and enzyme inhibitory activity. Targeted Targeted liquid chromatography–tandem mass spectrometry (LC–MS/MS) screening of 31 phenolics detected 23 compounds, with rosmarinic acid as the predominant constituent, reaching 19,650.23 µg/g extract in the water extract. The water extract also showed the highest extraction yield (11.14%), total phenolic content (71.62 mg gallic acid equivalents (GAE)/g extract), and activities in the phosphomolybdenum, 2,2-diphenyl-1-picrylhydrazyl (DPPH), 2,2′-azino-bis(3-ethylbenzothiazoline-6-sulfonic acid) (ABTS), cupric reducing antioxidant capacity (CUPRAC), and ferric reducing antioxidant power (FRAP) assays. In contrast, the methanol extract exhibited the highest total flavonoid content (17.73 mg RE/g extract) and the strongest tyrosinase and α-amylase inhibitory activities. Verbascoside was markedly enriched in the ethyl acetate extract (1288.60 µg/g extract), which also displayed the greatest metal-chelating activity. These findings provide the first comprehensive phenolic and bioactivity profile of *M. aybalae* and highlight extraction-condition-dependent differences in phenolic composition and in vitro activities, establishing a foundation for future investigations of this endemic species.

## 1. Introduction

Oxidative stress results from an imbalance between the generation of reactive oxygen species (ROS) and the capacity of endogenous antioxidant defense systems to neutralize them. Persistent oxidative imbalance can promote oxidative damage to lipids, proteins, and nucleic acids and has been associated with the development and progression of several chronic disorders, including cardiovascular diseases, neurodegenerative conditions, type 2 diabetes, and cancer. In this context, plant-derived phenolic compounds have attracted considerable interest as natural antioxidants because of their ability to scavenge reactive species, donate electrons or hydrogen atoms, chelate pro-oxidant transition metals, and modulate redox-related processes [1,2]. Phenolic acids and flavonoids, in particular, display diverse structure–activity relationships that contribute to their antioxidant and enzyme inhibitory properties [3,4].

Members of the Lamiaceae family represent an important source of phenolic compounds with antioxidant potential. Rosmarinic acid, caffeic acid, chlorogenic acid, phenylethanoid glycosides, and various flavonoids are frequently reported among the principal non-volatile constituents of this family [5]. However, the phytochemical composition and biological activity of plant extracts are strongly influenced by the extraction procedure. Solvent polarity, in particular, affects the solubility and recovery of chemically diverse phenolic constituents and can consequently alter the antioxidant and enzyme inhibitory properties of the resulting extracts. Nevertheless, extraction temperature, duration, and technique are also important determinants of phenolic recovery and stability, and their effects may interact with those of solvent properties [6,7]. Consequently, comparisons involving different solvents and extraction protocols should be interpreted in the context of the combined extraction conditions rather than solvent polarity alone. Comparative extraction approaches therefore provide a useful strategy for characterizing under-investigated plant species and identifying extraction-condition-dependent differences in phenolic composition and bioactivity [8,9].

The genus *Micromeria* Benth. (Lamiaceae, subfamily Nepetoideae, tribe Mentheae) comprises more than 130 species of shrubs, subshrubs, and perennial herbs distributed mainly throughout the Mediterranean Basin, Macaronesia, and southwestern Asia [3,4]. Several *Micromeria* species have traditionally been consumed as infusions or decoctions and used for gastrointestinal and respiratory complaints and other ethnomedicinal purposes [3,10]. Phytochemical investigations of the genus have repeatedly identified rosmarinic acid as a major phenolic constituent, together with variable amounts of caffeic acid, chlorogenic acid, verbascoside, and flavonoid derivatives [1,2,11]. Extracts of species including *M. nervosa*, *M. myrtifolia*, *M. fruticosa*, *M. frivaldszkyana*, *M. graeca*, *M. cymuligera*, and *M. croatica* have also demonstrated antioxidant and other biological activities, although considerable interspecific and solvent-dependent variation has been reported [12,13,14,15,16,17]. Despite these advances, several geographically restricted members of the genus remain poorly characterized with respect to their non-volatile phytochemistry.

*Micromeria aybalae* H.Duman & Dirmenci is a narrowly distributed endemic species originally described from southwestern Türkiye [18]. Although its essential oil composition and associated biological properties have recently been investigated [19], the quantitative distribution of its non-volatile phenolic constituents and the corresponding in vitro bioactivity profiles of different solvent extracts remain insufficiently characterized. Therefore, the present study aimed to comparatively characterize ethyl acetate, methanol, and water extracts of the aerial parts of *M. aybalae*. Extraction yields, total phenolic and flavonoid contents, and targeted LC–MS/MS profiles of 31 phenolic compounds were determined, together with antioxidant capacity evaluated using six complementary chemical assays and inhibitory activities against tyrosinase and α-amylase. The scientific novelty of this study lies in establishing the first integrated quantitative phenolic dataset for this endemic species and in identifying extraction-condition-dependent differences in the recovery of individual phenolic constituents, particularly rosmarinic acid, caffeic acid, and verbascoside. By combining compound-specific LC–MS/MS quantification with complementary in vitro activity measurements, the study provides a molecular-compositional framework for future mechanistic investigations, without claiming to establish the molecular mechanisms underlying the observed activities.

## 2. Results

### 2.1. Extraction Yield, Total Phenolic Content, Total Flavonoid Content, and RACI

The extraction yields, total phenolic content (TPC), total flavonoid content (TFC), and relative antioxidant capacity index (RACI) values of the ethyl acetate (EA), methanol (MeOH), and water extracts of *M. aybalae* are presented in Table 1. Extraction yield differed significantly among the solvents and followed the order water extract (11.14 ± 1.11%) > MeOH extract (2.45 ± 0.31%) > EA extract (1.45 ± 0.15%) (*p* < 0.05).

A similar pattern was observed for TPC. The water extract exhibited the highest TPC (71.62 ± 0.10 mg GAE/g extract), followed by the MeOH (32.48 ± 1.61 mg GAE/g extract) and EA (7.03 ± 0.25 mg GAE/g extract) extracts, with significant differences among all three extracts (*p* < 0.05). To further evaluate extraction efficiency relative to the initial dry plant material, the TPC values were adjusted using the corresponding mean extraction yields. The calculated TPC equivalents were highest for the water extract (7.98 mg GAE/g dry plant material), followed by the MeOH (0.80 mg GAE/g) and EA (0.10 mg GAE/g) extracts. These results represent yield-adjusted Folin–Ciocalteu assay equivalents rather than experimentally determined absolute recoveries of total phenolic compounds.

In contrast, TFC showed a different solvent-dependent pattern. The MeOH extract contained the highest TFC (17.73 ± 0.78 mg RE/g extract), significantly exceeding those of the EA (13.22 ± 0.90 mg RE/g extract) and water (10.18 ± 1.10 mg RE/g extract) extracts. No significant difference was observed between the EA and water extracts.

The RACI values further differentiated the integrated antioxidant-related profiles of the extracts, with the water extract showing the highest value (0.84 ± 0.06), followed by the MeOH (−0.22 ± 0.04) and EA (−0.62 ± 0.02) extracts (*p* < 0.05). Overall, the water extract was characterized by the highest extraction yield, phenolic content, and integrated antioxidant-related profile, whereas the MeOH extract was distinguished by its higher flavonoid content. Because RACI combines potentially correlated colorimetric content estimates and antioxidant assay responses, these values should be interpreted as descriptive composite scores rather than independent confirmation of differences in antioxidant capacity among the extracts.

### 2.2. LC–MS/MS Phenolic Profiling

Targeted LC–MS/MS analysis was performed to characterize 31 phenolic compounds in the EA, MeOH, and water extracts of *M. aybalae* (Table 2). Of the 31 compounds screened, 23 were detected in at least one extract, whereas pyrocatechol, 3-hydroxybenzoic acid, sinapic acid, 2-hydroxycinnamic acid, eriodictyol, quercetin, luteolin, and kaempferol were not detected in any extract. Importantly, the designation ND reflects non-detection within the analytical sensitivity of the employed method and does not necessarily imply that the corresponding compound is chemically absent from the extract.

Rosmarinic acid was the predominant quantified phenolic compound in all three extracts, with the highest concentration observed in the water extract (19,650.23 ± 199.54 µg/g extract), followed by the MeOH (11,380.19 ± 91.61 µg/g extract) and EA (380.45 ± 7.35 µg/g extract) extracts (*p* < 0.05). Caffeic acid showed a similar solvent-dependent pattern, reaching its highest level in the water extract (1188.49 ± 21.33 µg/g extract), compared with 320.21 ± 1.80 µg/g extract in MeOH and 42.97 ± 0.72 µg/g extract in EA.

In contrast, verbascoside was markedly enriched in the EA extract, where it reached 1288.60 ± 62.36 µg/g extract, compared with 184.46 ± 4.14 µg/g extract in the water extract and 155.74 ± 11.69 µg/g extract in the MeOH extract (*p* < 0.05). Chlorogenic acid also occurred at its highest concentration in the EA extract (522.59 ± 35.20 µg/g extract), followed by the water (109.34 ± 5.40 µg/g extract) and MeOH (25.82 ± 8.11 µg/g extract) extracts. Pinoresinol similarly predominated in EA (185.40 ± 6.22 µg/g extract).

Several other phenolics were preferentially recovered in the water extract. Protocatechuic acid (298.76 ± 1.73 µg/g extract), 3,4-dihydroxyphenylacetic acid (367.58 ± 3.13 µg/g extract), and syringic acid (172.74 ± 2.46 µg/g extract) reached their highest concentrations in this extract. By comparison, the MeOH extract contained the highest levels of apigenin 7-glucoside (162.61 ± 1.24 µg/g extract), luteolin 7-glucoside (35.02 ± 0.88 µg/g extract), and hyperoside (32.81 ± 1.77 µg/g extract).

Five compounds—(+)-catechin, (−)-epicatechin, vanillin, taxifolin, and apigenin—were detected exclusively in the MeOH extract, at 26.70 ± 3.31, 58.23 ± 0.40, 47.44 ± 5.61, 10.16 ± 0.25, and 51.97 ± 3.52 µg/g extract, respectively. In addition, 2,5-dihydroxybenzoic acid was detected only in the water extract (36.13 ± 3.34 µg/g extract).

To account for the differences in extraction yield, the phenolic concentrations were additionally recalculated relative to the initial dry plant material using the mean extraction yields (Supplementary Table S3). On this basis, the water extract showed the highest calculated rosmarinic acid content (2189.04 µg/g dry plant material), followed by the MeOH (278.81 µg/g) and EA (5.52 µg/g) extracts. The corresponding caffeic acid contents were 132.40, 7.85, and 0.62 µg/g dry plant material, respectively. Notably, although verbascoside was most concentrated in the EA extract when expressed per gram of extract, its calculated content per gram of initial dry plant material was slightly higher for the water extract (20.55 µg/g) than for the EA extract (18.68 µg/g). These values represent yield-adjusted estimates derived from group means rather than independently measured extraction recoveries.

### 2.3. Antioxidant Activity

The antioxidant activities of the *M. aybalae* extracts were evaluated using six complementary assays representing different antioxidant mechanisms (Table 3). Significant differences were observed among the extracts in all assays (*p* < 0.05).

The water extract exhibited the highest activity in all five redox-based assays, with values of 328.45 ± 8.86 mg TE/g extract for the phosphomolybdenum assay, 423.26 ± 5.97 mg TE/g extract for DPPH, 520.25 ± 35.61 mg TE/g extract for ABTS, 945.86 ± 5.70 mg TE/g extract for CUPRAC, and 530.03 ± 12.04 mg TE/g extract for FRAP. In each of these assays, the MeOH extract showed intermediate activity, whereas the EA extract exhibited the lowest values.

In contrast, the metal chelating activity showed a different pattern. The EA extract displayed the highest metal chelating activity (26.04 ± 0.19 mg EDTAE/g extract), followed by the water extract (22.50 ± 1.13 mg EDTAE/g extract) and the MeOH extract (11.38 ± 0.92 mg EDTAE/g extract), with significant differences among all three extracts (*p* < 0.05).

Overall, these results indicate a clear solvent-dependent variation in antioxidant performance, with the water extract showing the strongest radical-scavenging and reducing capacities, whereas the EA extract was characterized by superior metal chelating activity.

To facilitate comparison on the basis of the initial dry plant material, the antioxidant assay results were additionally adjusted for the corresponding mean extraction yields (Supplementary Table S4). The water extract exhibited the highest calculated yield-adjusted responses in all six assays, with values of 36.59, 47.15, 57.96, 105.37, and 59.05 mg TE/g dry plant material for phosphomolybdenum, DPPH, ABTS, CUPRAC, and FRAP, respectively, and 2.51 mg EDTAE/g dry plant material for metal chelation. Interestingly, although the EA extract showed the highest metal-chelating activity per gram of extract, the water extract provided the highest calculated metal-chelating equivalents per gram of initial dry plant material. These yield-adjusted values are descriptive estimates and should not be interpreted as absolute antioxidant recovery percentages.

### 2.4. Enzyme Inhibitory Activity

The enzyme inhibitory activities of the *M. aybalae* extracts against tyrosinase and α-amylase are presented in Table 3. Significant differences were observed among the three extracts for both enzyme assays (*p* < 0.05).

The MeOH extract exhibited the strongest tyrosinase inhibitory activity, with a value of 230.71 ± 2.84 mg KAE/g extract, followed by the water extract (169.19 ± 0.45 mg KAE/g extract) and the EA extract (116.16 ± 3.71 mg KAE/g extract).

A similar predominance of the MeOH extract was observed for α-amylase inhibition. The MeOH extract showed the highest activity (447.82 ± 7.61 mg ACE/g extract), followed by the EA extract (162.48 ± 2.65 mg ACE/g extract), whereas the water extract exhibited the lowest activity (47.32 ± 2.30 mg ACE/g extract).

To further characterize the inhibitory potency of the extracts, concentration–response experiments were performed, and IC_50_ values were determined (Table 4). The methanol extract exhibited the strongest tyrosinase inhibitory potency, with an IC_50_ value of 1.41 ± 0.02 mg/mL, followed by the water (1.92 ± 0.01 mg/mL) and ethyl acetate (2.79 ± 0.09 mg/mL) extracts. Kojic acid, used as the reference inhibitor, exhibited an IC_50_ value of 0.055 ± 0.001 mg/mL. Similarly, the methanol extract displayed the strongest α-amylase inhibition (IC_50_ = 2.31 ± 0.04 mg/mL), followed by the ethyl acetate (6.35 ± 0.10 mg/mL) and water (18.76 ± 0.95 mg/mL) extracts. The reference inhibitor acarbose exhibited an IC_50_ value of 1.05 ± 0.01 mg/mL. These results are consistent with the inhibitory activity rankings obtained using kojic acid and acarbose equivalents, providing complementary quantitative information on the inhibitory potency of the investigated extracts.

Overall, the MeOH extract was distinguished by its superior inhibitory activity against both tyrosinase and α-amylase.

## 3. Discussion

This study provides the first comprehensive evaluation of the phenolic composition, antioxidant capacity, and enzyme inhibitory activities of *M. aybalae* aerial part extracts, establishing a baseline phytochemical and bioactivity profile for this narrow endemic species. The principal scientific contribution is the quantitative characterization of individual phenolic constituents and their differential enrichment under the extraction conditions investigated. Rosmarinic acid is a widely distributed hydroxycinnamic acid derivative in the Lamiaceae family and has been extensively investigated for its antioxidant and other biological properties [20]. In this context, the high abundance of rosmarinic acid in the aqueous extract of *M. aybalae*, together with the contrasting distribution of caffeic acid and verbascoside among the extracts, provides new species-specific quantitative information. Importantly, these findings should be interpreted as evidence of differences in chemical composition and measured in vitro activities rather than direct evidence of compound-specific molecular mechanisms.

### 3.1. Solvent-Dependent Phenolic Composition and Antioxidant Capacity

The extraction yield varied markedly among the solvents and followed the order water extract > MeOH extract > EA extract. This pattern is consistent with the ability of water to recover a broad range of polar constituents from plant matrices. A similar solvent-dependent trend has been reported for other *Micromeria* species, including *M. myrtifolia*, in which polar extracts generally showed higher extraction yields and phenolic contents than less polar fractions [9]. In the present study, the water extract also exhibited the highest TPC, whereas the MeOH extract contained the highest TFC, indicating differences in phenolic and flavonoid recovery under the extraction conditions employed rather than demonstrating an effect attributable exclusively to solvent polarity.

An important consideration in interpreting these findings is the limited analytical specificity of the colorimetric assays used to estimate total phenolic content (TPC) and total flavonoid content (TFC). The Folin–Ciocalteu assay is based on electron-transfer reactions and may respond to non-phenolic reducing substances in addition to phenolic compounds [21]. Similarly, the aluminum chloride assay depends on the complexation properties of particular flavonoid structures and does not provide an equally sensitive response to all flavonoid subclasses [22]. Consequently, TPC and TFC should be interpreted as operationally defined assay equivalents rather than direct measurements of the absolute abundance of all phenolic compounds or flavonoids. Furthermore, the targeted LC–MS/MS analysis quantifies only the predefined analytes included in the analytical panel and therefore does not represent the complete phenolic composition of the extracts. Accordingly, differences between the colorimetric measurements and the targeted LC–MS/MS profiles should be interpreted in light of the distinct analytical principles and scopes of these methods rather than regarded as directly comparable quantitative measurements of total phenolic or flavonoid abundance.

Targeted LC–MS/MS analysis revealed rosmarinic acid as the predominant quantified phenolic in all three extracts. Its concentration reached 19,650.23 µg/g extract in the water extract and 11,380.19 µg/g extract in the MeOH extract, whereas substantially lower levels were detected in the EA extract. The predominance of rosmarinic acid is consistent with previous reports on *Micromeria* and related Lamiaceae taxa, in which this compound is frequently identified as one of the major phenolic constituents [4,5]. Rosmarinic acid has likewise been reported as a major phenolic constituent of *M. frivaldszkyana* and *M. cymuligera*, although direct quantitative comparison among studies is limited by differences in extraction procedures and the basis used to express phenolic concentrations [1,2].

Caffeic acid also showed pronounced enrichment in the water extract, reaching 1188.49 µg/g extract, together with elevated levels of protocatechuic acid and syringic acid. By contrast, verbascoside and chlorogenic acid were preferentially concentrated in the EA extract. Verbascoside was the most abundant quantified phenolic in the EA extract, reaching 1288.60 µg/g extract, while chlorogenic acid reached 522.59 µg/g extract. These contrasting distribution patterns highlight the influence of the extraction protocols on the recovery of individual phenolic constituents rather than simply reflecting differences in total phenolic yield. However, the individual contributions of solvent polarity, extraction temperature, and extraction duration cannot be distinguished using the present experimental design.

Importantly, the differences observed among the extracts should be interpreted in relation to the combined extraction conditions. In the present investigation, ethyl acetate and methanol extraction were performed by maceration at room temperature for 24 h, whereas the aqueous extract was prepared by infusion using boiling water for 15 min. Previous studies have established that phenolic extraction efficiency depends not only on solvent properties but also on extraction temperature, duration, and the characteristics of the plant matrix [6,7]. Therefore, the higher recovery of rosmarinic acid and caffeic acid and the greater antioxidant capacities observed in the aqueous extract cannot be attributed exclusively to the polarity of water. These findings instead reflect the combined effects of the specific extraction conditions employed. Further investigations using standardized extraction temperatures and durations across different solvents would be required to distinguish solvent-specific effects from those associated with extraction conditions.

The antioxidant activity data closely reflected these solvent-dependent differences. The water extract exhibited the highest activity in the phosphomolybdenum, DPPH, ABTS, CUPRAC, and FRAP assays. Its superior performance in these redox-based assays was consistent with its high TPC and the marked accumulation of rosmarinic acid, caffeic acid, protocatechuic acid, and syringic acid. Similar relationships between phenolic richness and radical-scavenging or reducing capacity have been described for *M. graeca*, *M. frivaldszkyana*, and *M. fruticosa* subsp. *brachycalyx* [2,14,16]. These observations are also consistent with reports from other Lamiaceae taxa in which phenolic-rich extracts exhibited stronger redox-based antioxidant activity [23,24].

In contrast to the redox-based assays, metal chelating activity followed the order EA extract > water extract > MeOH extract. This distinct pattern suggests that metal chelation was influenced by the qualitative composition of the extracts rather than by TPC alone. The relatively high levels of verbascoside, chlorogenic acid, and pinoresinol in the EA extract may have contributed to this response; however, the present data do not establish the individual contribution of these compounds to the observed activity.

The MeOH extract displayed the strongest inhibition of both tyrosinase and α-amylase, despite having lower TPC and redox-based antioxidant activity than the water extract. This extract was characterized by the highest TFC and contained comparatively high levels of apigenin 7-glucoside, luteolin 7-glucoside, hyperoside, and several compounds detected exclusively in MeOH. Although the MeOH extract exhibited the highest TFC and the strongest tyrosinase and α-amylase inhibitory activities, this co-occurrence does not establish a causal relationship between flavonoid abundance and enzyme inhibition. The specific contributions of individual phenolic constituents, including flavonoid derivatives, remain undetermined and would require direct testing of isolated compounds or fractionated extracts. Similar solvent-dependent differences in enzyme inhibitory activity have been reported for *M. cymuligera* and *M. myrtifolia* [1,9]. The concentration–response analyses further confirmed the greater inhibitory potency of the MeOH extract, which exhibited the lowest IC_50_ values against both tyrosinase (1.41 ± 0.02 mg/mL) and α-amylase (2.31 ± 0.04 mg/mL). These findings complement the reference-standard-equivalent activity measurements and provide a more directly comparable quantitative assessment of enzyme inhibitory potency under the experimental conditions employed.

An additional consideration in comparing the extraction protocols is the distinction between constituent concentrations or assay responses expressed per gram of extract and their corresponding yield-adjusted values expressed per gram of initial dry plant material. The supplementary calculations demonstrated that differences in extraction yield can alter the relative interpretation of extract performance. For instance, although the EA extract contained the highest concentration of verbascoside and exhibited the strongest metal-chelating activity on an extract-mass basis, the aqueous extraction protocol provided slightly higher calculated verbascoside content and greater metal-chelating equivalents when normalized to the initial dry plant material. In contrast, the aqueous protocol maintained its predominance in rosmarinic acid and caffeic acid contents and in the redox-based antioxidant assays under both expression bases. These comparisons emphasize the importance of considering extraction yield when evaluating the practical recovery of phenolic constituents and antioxidant assay equivalents. Nevertheless, the calculations are based on mean extraction yields and do not distinguish the independent contributions of solvent polarity, extraction temperature, or extraction duration.

### 3.2. Potential Contributions of Major Phenolic Compounds to the Observed Bioactivities

The observed antioxidant and enzyme inhibitory profiles of *M. aybalae* extracts can be discussed in the context of their quantified phenolic composition, particularly the distribution of rosmarinic acid, caffeic acid, chlorogenic acid, and verbascoside. However, the present study did not include experiments with isolated compounds or fractionated extracts, and therefore the individual contributions of these constituents to the measured activities cannot be determined. Accordingly, the following discussion is intended to provide literature-based chemical context for the observed extract-level activities rather than to establish compound-specific effects or causal structure–activity relationships.

Rosmarinic acid was the predominant quantified phenolic in all three extracts and was especially abundant in the water and MeOH extracts. Structurally, rosmarinic acid contains two catechol moieties that can support hydrogen atom donation, electron transfer, and metal-binding interactions, mechanisms that are consistent with antioxidant responses measured by DPPH, ABTS, CUPRAC, FRAP, and phosphomolybdenum assays [11]. Its high concentration in the water extract is therefore consistent with the strong redox-based antioxidant activity of this extract. Previous studies have also associated rosmarinic acid with enzyme inhibitory effects, including inhibition of tyrosinase and α-amylase, although such mechanisms were not directly examined in the present study [1,25].

Caffeic acid was most abundant in the water extract, which also exhibited the strongest responses in the redox-based antioxidant assays. Its catechol structure favors both hydrogen atom transfer and single-electron transfer reactions, which are relevant to radical-scavenging and reducing assays [16]. The co-occurrence of caffeic acid and rosmarinic acid in the water extract provides relevant compositional context for its strong responses in redox-based antioxidant assays; however, their individual contributions to these responses were not experimentally determined.

Chlorogenic acid showed a contrasting distribution and was most abundant in the EA extract. This compound has been reported to possess both antioxidant and α-amylase inhibitory properties [1]. Although chlorogenic acid was more abundant in the EA extract, the MeOH extract exhibited substantially stronger α-amylase inhibition. This observation indicates that the distribution of chlorogenic acid alone does not explain the differences in inhibitory activity among the extracts. However, the contribution of chlorogenic acid to the measured activity cannot be established without direct experimental evaluation of the isolated compound.

Verbascoside was the most abundant quantified phenolic in the EA extract. Owing to its phenylethanoid glycoside structure and multiple phenolic hydroxyl groups, verbascoside has been associated with antioxidant and metal-binding properties [2,4]. Its enrichment in the EA extract, together with the relatively high levels of chlorogenic acid and pinoresinol, may be consistent with the superior metal chelating activity observed for this extract. Nevertheless, the present findings do not permit attribution of this activity to any single compound.

Taken together, the quantified phenolic profiles revealed distinct compositional differences among the extracts, which were accompanied by differences in their measured antioxidant and enzyme inhibitory activities. The water extract contained the highest concentrations of rosmarinic and caffeic acids and exhibited the strongest responses in the redox-based antioxidant assays, whereas the EA extract was enriched in verbascoside and chlorogenic acid and showed the highest metal-chelating activity. In contrast, the MeOH extract exhibited the strongest tyrosinase and α-amylase inhibition, including the lowest IC_50_ values among the investigated extracts. Although these patterns are consistent with previously reported chemical and biological properties of certain phenolic constituents, the present findings do not establish which compounds, or combinations of compounds, are responsible for the observed activities. Such relationships require direct experimental verification using isolated compounds, fractionated extracts, or appropriately designed bioactivity-guided investigations.

### 3.3. Comparison with Other Micromeria Species

The phytochemical profile of *M. aybalae* shows several similarities with previously investigated *Micromeria* species, particularly with respect to the predominance of rosmarinic acid. Rosmarinic acid has repeatedly been identified as a major phenolic constituent in *M. cymuligera*, *M. frivaldszkyana*, *M. nervosa*, and *M. myrtifolia*, supporting its importance as a recurrent phytochemical feature of the genus [1,2,12,13]. Nevertheless, direct quantitative comparisons among studies should be interpreted cautiously because reported values are influenced by differences in plant material, geographic origin, phenological stage, solvent system, extraction procedure, analytical platform, and the expression basis.

In the present study, rosmarinic acid was the dominant quantified phenolic in all three *M. aybalae* extracts and reached its highest concentration in the water extract. This finding is consistent with the rosmarinic acid-rich profiles previously documented in *M. frivaldszkyana*, *M. nervosa*, *M. myrtifolia*, and *M. cymuligera* [1,2,12,13]. Caffeic acid, a biosynthetically related hydroxycinnamic acid, was also markedly enriched in the water extract. Previous studies on *M. frivaldszkyana*, *M. nervosa*, *M. myrtifolia*, and *M. cymuligera* have likewise reported caffeic acid in varying amounts, indicating substantial species- and solvent-dependent variation [1,2,12,13].

Chlorogenic acid also displayed a distinct solvent-dependent distribution in *M. aybalae*, with the highest concentration observed in the EA extract. Considerable variation in chlorogenic acid abundance has previously been reported among *Micromeria* species, including *M. frivaldszkyana*, *M. nervosa*, *M. myrtifolia*, and *M. cymuligera* [1,2,12,13]. This variability is consistent with the broader phytochemical heterogeneity of the genus, in which the relative abundance of hydroxycinnamic acids, flavonoid derivatives, and other phenolic subclasses differs considerably among species and extraction systems [4,5].

A particularly notable finding in *M. aybalae* was the pronounced accumulation of verbascoside in the EA extract. Verbascoside has previously been reported in *Micromeria* taxa, including *M. nervosa* and *M. myrtifolia* [12,13], whereas it was not detected in the reported phenolic profile of *M. cymuligera* [1]. The comparatively high abundance observed in the EA extract of *M. aybalae* may therefore represent a notable feature of its phenolic profile. However, because only one population and one collection period were examined in the present study, additional populations and seasonal sampling would be required to determine the consistency of this pattern.

The solvent-dependent antioxidant profile observed in *M. aybalae* is also broadly consistent with previous studies on the genus. Solvent-related differences in radical-scavenging and reducing activities have been reported for *M. graeca*, *M. frivaldszkyana*, *M. fruticosa* subsp. *brachycalyx*, *M. nervosa*, and *M. myrtifolia* [2,12,13,14,16,26]. In the present study, the strong redox-based antioxidant activity of the water extract paralleled its high TPC and abundance of rosmarinic and caffeic acids. By contrast, the EA extract exhibited the highest metal chelating activity, whereas the MeOH extract showed the strongest tyrosinase and α-amylase inhibition. These differences further indicate that individual biological activities cannot be explained solely by total phenolic content.

Taken together, these comparisons indicate that *M. aybalae* shares the rosmarinic acid-rich phytochemical pattern frequently observed within the genus, while displaying a distinct solvent-dependent distribution of caffeic acid, chlorogenic acid, verbascoside, and flavonoid derivatives. The present findings therefore place *M. aybalae* within the broader phytochemical context of *Micromeria* while also highlighting features that merit further investigation across different populations, collection periods, and extraction systems.

### 3.4. Functional Relevance and Health Implications

The phenolic-rich profile and antioxidant capacity of *M. aybalae* extracts suggest potential relevance to the broader field of plant-derived antioxidants and oxidative stress-related research. In particular, the marked enrichment of rosmarinic and caffeic acids in the water extract is noteworthy, as both compounds have been widely investigated for their antioxidant and anti-inflammatory properties and their capacity to modulate cellular processes associated with oxidative stress. Rosmarinic acid has been reported to influence oxidative and inflammatory signaling pathways, including Nrf2- and NF-κB-related mechanisms, while caffeic acid and its derivatives have demonstrated antioxidant and anti-inflammatory effects in a range of preclinical models [27,28]. The strong activity of the water extract across DPPH, ABTS, CUPRAC, FRAP, and phosphomolybdenum assays is therefore chemically consistent with its high abundance of these redox-active phenolics. However, the present assays represent cell-free antioxidant models and do not demonstrate modulation of these signaling pathways by *M. aybalae* itself. It is important to distinguish the chemical antioxidant capacity measured in the present study from antioxidant protection in biological systems. Conventional antioxidant assays, including DPPH, ABTS, CUPRAC, and FRAP, primarily characterize the radical-scavenging or reducing properties of samples under defined chemical reaction conditions [29,30]. However, antioxidant effectiveness in biological systems also depends on factors such as cellular uptake, metabolism, localization, and interactions with endogenous antioxidant defense mechanisms [31]. Therefore, the strong antioxidant responses observed for the aqueous extract of *M. aybalae* should be interpreted as evidence of chemical antioxidant capacity rather than cellular protection against oxidative stress.

Several phenolic compounds identified in the investigated extracts, including rosmarinic acid, caffeic acid, chlorogenic acid, and verbascoside, have been associated with antioxidant, anti-inflammatory, metabolic, or neuroprotective properties in previous experimental studies [27,28,32,33]. However, these literature-reported properties concern individual compounds or other experimental systems and should not be extrapolated to the *M. aybalae* extracts investigated here. The present study did not evaluate cellular signaling pathways, metabolic regulation, neuroprotection, or anti-inflammatory activity, and therefore no conclusions regarding these biological effects or potential therapeutic benefits can be drawn from the current results.

The pronounced tyrosinase and α-amylase inhibitory activities of the MeOH extract further indicate that the functional properties of *M. aybalae* cannot be explained solely by TPC or antioxidant capacity. However, the present findings do not establish whether the observed inhibitory activities arise from individual phenolic acids, flavonoid derivatives, other extract constituents, or potential interactions among them. Accordingly, attributing the stronger enzyme inhibitory activity of the MeOH extract to its flavonoid-enriched composition would remain speculative without direct experimental verification. From a functional perspective, these findings identify *M. aybalae* as a promising subject for further investigation rather than demonstrating a direct health benefit. Future studies employing standardized extracts, isolated compounds, cell-based oxidative stress and inflammation models, bioavailability assessments, and in vivo approaches will therefore be necessary to determine whether the chemical and cell-free activities observed here translate into biologically relevant effects.

### 3.5. Study Limitations and Future Perspectives

The present study has several methodological and experimental limitations that should be considered when interpreting the findings. Although the quantitative phenolic profiles and in vitro activities revealed differences among the three extraction protocols, the limited number of extract types and the absence of independent biological samples precluded robust statistical evaluation of relationships between individual phenolic compounds and the measured activities. Consequently, no compound-specific structure–activity relationships or causal associations can be established from the present dataset. Future studies involving independent biological samples, a broader range of extraction conditions, and direct testing of isolated compounds would be necessary to investigate such relationships more reliably.

An important methodological limitation concerns the differences in extraction conditions among the investigated solvents. Although the same plant-to-solvent ratio was used, the organic extracts were prepared by maceration at room temperature for 24 h, whereas the aqueous extract was obtained by a 15 min infusion using boiling water. Consequently, solvent type was not independently varied under otherwise identical extraction conditions, and the effects of solvent polarity cannot be separated from those of extraction temperature, duration, and technique. Therefore, the observed differences in extraction yield, phenolic composition, and in vitro activities should be interpreted as extraction-protocol-dependent rather than exclusively solvent-polarity-dependent. A controlled factorial experimental design would be necessary to determine the independent and interactive effects of these extraction parameters.

Another limitation is that the LC–MS/MS analysis was based on a targeted panel of 31 predefined phenolic compounds. Targeted analytical approaches provide sensitive and quantitative measurement of selected analytes but inherently restrict detection to compounds included in the predefined analytical panel; consequently, other potentially relevant metabolites may remain uncharacterized [34]. Untargeted metabolomic approaches would therefore be valuable for expanding the chemical characterization of *M. aybalae*.

In addition, the plant material was collected from a single natural population during one sampling period, and all extractions were performed using aliquots of one pooled and homogenized sample. Although three independent extraction procedures were conducted for each solvent, these replicates reflect variability associated with the extraction process rather than biological variation among individual plants, populations, seasons, or developmental stages. Consequently, the findings should be considered specific to the investigated plant material and extraction conditions, and their generalizability to other populations or collection periods remains uncertain. Future investigations involving independently collected biological samples from different populations, seasons, and developmental stages are necessary to establish the broader reproducibility of the observed phytochemical and bioactivity patterns.

Furthermore, the antioxidant activities were evaluated using cell-free chemical assays. Although these assays are useful for characterizing radical-scavenging, reducing, and metal-chelating properties under defined experimental conditions, they do not reproduce the complexity of biological systems. Therefore, the measured antioxidant capacities cannot be directly extrapolated to cellular, in vivo, or clinical efficacy [35]. Further investigations using appropriate cell-based oxidative stress models, together with assessments of cytotoxicity, intracellular reactive oxygen species generation, and endogenous antioxidant defense responses, are necessary to determine whether the chemical antioxidant properties observed in the present study translate into biologically relevant protective effects. The enzyme inhibitory evaluation was also restricted to tyrosinase and α-amylase, and other potentially relevant enzymatic targets were not investigated.

Finally, the present investigation was designed to characterize phenolic composition and evaluate chemical antioxidant and enzyme inhibitory activities rather than to elucidate specific molecular mechanisms. No experiments involving isolated phenolic compounds, cellular signaling pathways, gene expression, or direct molecular target interactions were conducted. Consequently, the observed differences in phenolic composition and in vitro activities cannot establish causal or mechanistic relationships between individual constituents and the measured responses. Nevertheless, the quantitative phenolic dataset generated in this study provides a foundation for future investigations using purified compounds, bioactivity-guided fractionation, cell-based assays, and molecular-level approaches to determine the specific contributions and mechanisms of the identified constituents.

## 4. Materials and Methods

### 4.1. Plant Material and Extraction

The aerial parts of *Micromeria aybalae* H.Duman & Dirmenci were collected on 1 November 2023 from Sandras Mountain, Köyceğiz district, Muğla Province, southwestern Türkiye (37°0′22″ N, 28°48′44″ E; 940 m a.s.l.). The species was observed growing naturally on rocky, calcareous soils at a south-facing site exposed to direct sunlight. The plant material was authenticated by Dr. Olcay Ceylan (Department of Biology, Muğla Sıtkı Koçman University, Türkiye). A herbarium specimen was prepared from the collected material and assigned the reference number O.2188.All chemicals, reagents, authentic reference standards used for LC–MS/MS analysis, and enzymes employed in this study were purchased from Sigma-Aldrich (St. Louis, MO, USA). 

The collected aerial parts were air-dried in the shade at room temperature and subsequently ground. All dried plant materials collected from the same population during a single sampling period were combined, ground, and thoroughly homogenized to obtain one pooled plant sample. This pooled sample served as the common starting material for all subsequent extractions. For the preparation of the ethyl acetate (EA) and methanol (MeOH) extracts, the powdered plant material was extracted with the respective solvent at a plant-to-solvent ratio of 1:20 (g/mL) by maceration at room temperature for 24 h. Following extraction, the mixtures were filtered, and the solvents were removed under reduced pressure using a rotary evaporator.

For the water extract, powdered plant material was mixed with boiling distilled water at the same 1:20 (g/mL) ratio and subjected to infusion for 15 min. The mixture was subsequently filtered, and the resulting water extract was lyophilized. For each solvent, three independent extraction procedures were performed using separate aliquots of the same pooled and homogenized plant material (*n* = 3). Thus, these replicates represented independent extraction preparations rather than independent biological samples from different plants or populations.

### 4.2. Phytochemical Analysis

Total phenolic content (TPC) and total flavonoid content (TFC) were determined using the Folin–Ciocalteu and aluminum chloride colorimetric methods, respectively, and expressed as mg gallic acid equivalents (GAE)/g extract and mg rutin equivalents (RE)/g extract [36]. Detailed experimental procedures are provided in Supplementary Materials, Section S.1. Because these colorimetric assays are not entirely specific to phenolic compounds or flavonoids, the resulting TPC and TFC values were interpreted as gallic acid and rutin equivalents, respectively, rather than as absolute concentrations of the corresponding compound classes.

Phenolic profiling was performed using a previously validated LC–ESI–MS/MS method with an Agilent 1260 Infinity LC system (Agilent Technologies, Santa Clara, CA, USA) coupled to a 6420 Triple Quad mass spectrometer (Agilent Technologies, Santa Clara, CA, USA). Chromatographic separation was achieved on a Poroshell 120 EC-C18 column, and analytes were quantified using authentic standards in multiple reaction monitoring (MRM) mode [37]. The original method validation, performed using an olive leaf matrix, reported analytical recovery values ranging from 78.1% to 108.7% and included intra-day and inter-day precision assessments. These published validation results should not be interpreted as matrix-specific recovery or precision measurements established for the *M. aybalae* extracts investigated in the present study. Analytes yielding signals below their compound-specific limits of detection (LOD) were reported as not detected (ND). Detailed analytical conditions, identification criteria, calibration characteristics, and compound-specific LOD and limits of quantification (LOQ) are provided in Supplementary Materials, Section S.1, Tables S1 and S2.

For comparison of phenolic contents relative to the initial dry plant material, the mean concentration of each phenolic compound (µg/g extract) was multiplied by the corresponding mean extraction yield expressed as a decimal fraction. The extraction yield factors were 0.1114, 0.0245, and 0.0145 for the water, MeOH, and EA extracts, respectively. The resulting values were expressed as µg/g initial dry plant material and are presented in Supplementary Table S3. The same yield-adjustment procedure was applied to the mean TPC values (mg GAE/g extract) to obtain estimates expressed as mg GAE/g initial dry plant material. Because these calculations were based on group means, the derived estimates are presented without propagated standard deviations or inferential statistical comparisons.

### 4.3. Determination of Antioxidant Activity

The antioxidant properties of the extracts were evaluated using six complementary assays: phosphomolybdenum, DPPH radical scavenging, ABTS radical cation scavenging, CUPRAC, FRAP, and ferrous ion chelating assays [38,39,40,41,42]. Antioxidant activities were expressed as mg Trolox equivalents (TE)/g extract, except for metal chelating activity, which was expressed as mg EDTA equivalents (EDTAE)/g extract. Detailed assay procedures are provided in Supplementary Materials, Section S.1. To estimate antioxidant assay responses relative to the initial dry plant material, the mean standard-equivalent activity values (mg TE/g extract or mg EDTAE/g extract) were multiplied by the corresponding mean extraction yields expressed as decimal fractions. The calculated results were reported as mg TE/g initial dry plant material for the five redox-based assays and mg EDTAE/g initial dry plant material for the metal-chelating assay (Supplementary Table S4). These yield-adjusted values provide descriptive estimates of assay equivalents per gram of starting plant material rather than experimentally determined recovery percentages of antioxidant compounds.

### 4.4. Determination of Enzyme Inhibitory Activity

The enzyme inhibitory activities of the extracts were evaluated against tyrosinase and α-amylase using spectrophotometric methods [36,43]. Tyrosinase inhibitory activity was determined using L-DOPA as substrate and expressed as mg kojic acid equivalents (KAE)/g extract, whereas α-amylase inhibitory activity was assessed using the Caraway–Somogyi iodine/potassium iodide method and expressed as mg acarbose equivalents (ACE)/g extract. The standard-equivalent inhibitory activities were determined by relating the blank-corrected assay responses of the extracts to the corresponding calibration curves of the reference inhibitors (kojic acid for tyrosinase and acarbose for α-amylase). The resulting activities were expressed as milligrams of reference-standard equivalents per gram of dry extract (mg KAE/g extract and mg ACE/g extract, respectively). These standard-equivalent values represent assay-specific comparative responses and should not be interpreted as direct measures of inhibitory potency or IC_50_. In addition to the reference-standard-equivalent activity measurements, concentration–response experiments were conducted to determine the half-maximal inhibitory concentration (IC_50_) values of the extracts against tyrosinase and α-amylase. Extract concentrations ranging from 0.5 to 20 mg/mL were evaluated, and IC_50_ values were calculated by nonlinear regression analysis of the concentration–response data. Kojic acid and acarbose were used as reference inhibitors for tyrosinase and α-amylase, respectively. The resulting IC_50_ values were expressed as mg/mL, with lower values indicating greater inhibitory potency. Detailed experimental conditions are described in Supplementary Materials, Section S.1.

### 4.5. Relative Antioxidant Capacity Index and Statistical Analysis

For each extraction procedure, three independently prepared extracts were obtained from separate aliquots of a single pooled and homogenized plant sample. Results are presented as mean ± standard deviation (SD) (*n* = 3), representing variability among independent extraction preparations under the conditions investigated. These replicates were independent at the extraction level but did not represent independent biological samples or populations. Differences among the three extract groups were evaluated using one-way analysis of variance (ANOVA), followed by Tukey’s honestly significant difference (HSD) post hoc test, with statistical significance set at *p* < 0.05. Accordingly, the statistical comparisons were restricted to the extraction preparations derived from the investigated pooled plant material and should not be interpreted as evidence of biological variability or population-level differences [44,45].

The relative antioxidant capacity index (RACI) was calculated to provide an integrated assessment of the antioxidant-related phytochemical and activity profile of the extracts. The index included eight parameters: phosphomolybdenum, DPPH, ABTS, CUPRAC, FRAP, metal-chelating activity, total phenolic content (TPC), and total flavonoid content (TFC). For each antioxidant assay, as well as TPC and TFC, the raw values were standardized as z-scores using the corresponding mean and standard deviation. The RACI value for each extract was then calculated as the mean of the standardized values obtained across all included parameters [46].

The inclusion of TPC and TFC alongside the six antioxidant assays was intended to provide a descriptive overview integrating operational estimates of phenolic and flavonoid content with complementary measures of in vitro antioxidant-related activity. However, these parameters represent different analytical properties and cannot be considered independent or interchangeable measures of antioxidant capacity. Furthermore, correlations among the included variables, particularly those reflecting overlapping electron-transfer or reducing mechanisms, may disproportionately influence the composite index and its ranking of extracts. Therefore, RACI was used exclusively as a descriptive summary of the selected standardized measurements and was not considered an independent validation of antioxidant superiority or evidence of distinct biological mechanisms.

## 5. Conclusions

This study provides the first comprehensive characterization of the phenolic composition, antioxidant capacity, and enzyme inhibitory activities of *M. aybalae* extracts prepared under three different extraction protocols. Among the three extracts, the water extract exhibited the highest extraction yield, total phenolic content, RACI value, and activity in all five redox-based antioxidant assays, whereas the MeOH extract showed the highest total flavonoid content and the strongest tyrosinase and α-amylase inhibitory activities. The EA extract was distinguished by the highest metal chelating activity.

Targeted LC–MS/MS analysis detected 23 of the 31 phenolic compounds investigated, with rosmarinic acid as the predominant constituent in all three extracts and reaching its highest concentration in the water extract. Caffeic acid was likewise enriched in the water extract, whereas verbascoside and chlorogenic acid were preferentially concentrated in the EA extract. These extraction-protocol-dependent differences in phenolic composition were accompanied by contrasting antioxidant and enzyme inhibitory profiles among the extracts. However, the observed associations do not establish causal relationships between individual phenolic constituents and the measured in vitro activities.

Overall, this study establishes a species-specific quantitative phytochemical and in vitro bioactivity baseline for *M. aybalae*, characterized by a rosmarinic acid-rich phenolic profile and extraction-protocol-dependent differences in the distribution of other phenolic constituents, particularly caffeic acid and verbascoside. The strong redox-based antioxidant capacity of the water extract identifies this extract as a subject of interest for further phytochemical and biological investigation. However, because the present findings are based on targeted chemical profiling and cell-free in vitro assays, they should not be interpreted as evidence of therapeutic efficacy. Furthermore, the use of a single pooled plant sample from one population and collection period limits the generalizability of the findings. Future studies incorporating untargeted metabolomics, cell-based models, bioavailability assessment, independent biological sampling across multiple populations and collection periods, and in vivo validation are warranted to further clarify the biological relevance and potential functional applications of this endemic species.

## Figures and Tables

**Table 1:** Extraction yield, total phenolic content (TPC), total flavonoid content (TFC), and relative antioxidant capacity index (RACI) of *M. aybalae* extracts.

Parameter	Ethyl Acetate	Methanol	Water
Yield (%)	1.45 ± 0.15 ^c^	2.45 ± 0.31 ^b^	11.14 ± 1.11 ^a^
Total phenolic (mg GAE/g extract)	7.03 ± 0.25 ^c^	32.48 ± 1.61 ^b^	71.62 ± 0.10 ^a^
Total flavonoid (mg RE/g extract)	13.22 ± 0.90 ^b^	17.73 ± 0.78 ^a^	10.18 ± 1.10 ^b^
RACI	−0.62 ± 0.02 ^c^	−0.22 ± 0.04 ^b^	0.84 ± 0.06 ^a^

Values are expressed as mean ± SD of three independent extraction preparations (*n* = 3) obtained from a single pooled and homogenized plant sample. These replicates do not represent independent biological samples. Different superscript letters within the same row indicate statistically significant differences among extraction preparations according to one-way ANOVA followed by Tukey’s HSD test (*p* < 0.05). GAE, gallic acid equivalents; RE, rutin equivalents; RACI, relative antioxidant capacity index.

**Table 2:** Targeted LC–MS/MS profiling of phenolic compounds in *M. aybalae* extracts (µg/g extract).

Compound	Ethyl Acetate	Methanol	Water
Gallic acid	ND	22.63 ± 0.67 ^a^	4.98 ± 0.02 ^b^
Protocatechuic acid	98.29 ± 4.53 ^c^	131.05 ± 0.53 ^b^	298.76 ± 1.73 ^a^
3,4-Dihydroxyphenylacetic acid	ND	31.36 ± 0.15 ^b^	367.58 ± 3.13 ^a^
(+)-Catechin	ND	26.70 ± 3.31	ND
Pyrocatechol	ND	ND	ND
Chlorogenic acid	522.59 ± 35.20 ^a^	25.82 ± 8.11 ^c^	109.34 ± 5.40 ^b^
2,5-Dihydroxybenzoic acid	ND	ND	36.13 ± 3.34
4-Hydroxybenzoic acid	ND	49.34 ± 1.72 ^a^	34.05 ± 0.64 ^b^
(−)-Epicatechin	ND	58.23 ± 0.40	ND
Vanillic acid	ND	101.94 ± 9.47 ^a^	74.62 ± 6.29 ^b^
Caffeic acid	42.97 ± 0.72 ^c^	320.21 ± 1.80 ^b^	1188.49 ± 21.33 ^a^
Syringic acid	ND	98.78 ± 3.86 ^b^	172.74 ± 2.46 ^a^
3-Hydroxybenzoic acid	ND	ND	ND
Vanillin	ND	47.44 ± 5.61	ND
Verbascoside	1288.60 ± 62.36 ^a^	155.74 ± 11.69 ^c^	184.46 ± 4.14 ^b^
Taxifolin	ND	10.16 ± 0.25	ND
Sinapic acid	ND	ND	ND
*p*-Coumaric acid	ND	6.97 ± 0.23 ^b^	9.36 ± 0.06 ^a^
Ferulic acid	ND	26.40 ± 2.12 ^a^	27.78 ± 0.19 ^a^
Luteolin 7-glucoside	ND	35.02 ± 0.88 ^a^	6.34 ± 0.44 ^b^
Hesperidin	ND	39.11 ± 3.18 ^a^	40.32 ± 2.24 ^a^
Hyperoside	ND	32.81 ± 1.77 ^a^	3.88 ± 0.04 ^b^
Rosmarinic acid	380.45 ± 7.35 ^c^	11,380.19 ± 91.61 ^b^	19,650.23 ± 199.54 ^a^
Apigenin 7-glucoside	ND	162.61 ± 1.24 ^a^	43.68 ± 0.07 ^b^
2-Hydroxycinnamic acid	ND	ND	ND
Pinoresinol	185.40 ± 6.22 ^a^	40.56 ± 7.02 ^b^	12.25 ± 0.11 ^c^
Eriodictyol	ND	ND	ND
Quercetin	ND	ND	ND
Luteolin	ND	ND	ND
Kaempferol	ND	ND	ND
Apigenin	ND	51.97 ± 3.52	ND

Values are expressed as mean ± SD of three independent extraction preparations (*n* = 3) obtained from a single pooled and homogenized plant sample. These replicates do not represent independent biological samples. Different superscript letters within the same row indicate statistically significant differences among extraction preparations according to one-way ANOVA followed by Tukey’s HSD test (*p* < 0.05). ND, not detected. Where a compound was detected in only one extract, no statistical comparison was performed.

**Table 3:** Antioxidant and enzyme inhibitory activities of *M. aybalae* extracts expressed as standard equivalent activities.

Assay	Unit	Ethyl Acetate	Methanol	Water
Phosphomolybdenum	mg TE/g extract	202.10 ± 6.65 ^c^	244.54 ± 3.54 ^b^	328.45 ± 8.86 ^a^
DPPH	mg TE/g extract	15.63 ± 1.76 ^c^	129.70 ± 1.63 ^b^	423.26 ± 5.97 ^a^
ABTS	mg TE/g extract	32.63 ± 3.46 ^c^	152.69 ± 2.77 ^b^	520.25 ± 35.61 ^a^
CUPRAC	mg TE/g extract	84.56 ± 3.39 ^c^	314.33 ± 6.77 ^b^	945.86 ± 5.70 ^a^
FRAP	mg TE/g extract	16.58 ± 0.21 ^c^	180.36 ± 5.88 ^b^	530.03 ± 12.04 ^a^
Metal chelating	mg EDTAE/g extract	26.04 ± 0.19 ^a^	11.38 ± 0.92 ^c^	22.50 ± 1.13 ^b^
Tyrosinase	mg KAE/g extract	116.16 ± 3.71 ^c^	230.71 ± 2.84 ^a^	169.19 ± 0.45 ^b^
α-Amylase	mg ACE/g extract	162.48 ± 2.65 ^b^	447.82 ± 7.61 ^a^	47.32 ± 2.30 ^c^

Values are expressed as mean ± SD of three independent extraction preparations (*n* = 3) obtained from a single pooled and homogenized plant sample. These replicates do not represent independent biological samples. Different superscript letters within the same row indicate statistically significant differences among extraction preparations according to one-way ANOVA followed by Tukey’s HSD test (*p* < 0.05). TE, Trolox equivalents; EDTAE, EDTA equivalents; KAE, kojic acid equivalents; ACE, acarbose equivalents.

**Table 4:** IC_50_ values (mg/mL) of *M. aybalae* extracts against tyrosinase and α-amylase.

Sample	Tyrosinase	α-Amylase
Ethyl acetate	2.79 ± 0.09 ^d^	6.35 ± 0.10 ^c^
Methanol	1.41 ± 0.02 ^b^	2.31 ± 0.04 ^b^
Water	1.92 ± 0.01 ^c^	18.76 ± 0.95 ^d^
Standard	0.055 ± 0.001 ^a^	1.05 ± 0.01 ^a^

Values are expressed as mean ± SD (*n* = 3). For plant extracts, the replicate values were obtained from three independent extraction preparations derived from a single pooled and homogenized plant sample and do not represent independent biological samples. Reference inhibitors were measured separately. Different superscript letters within the same column indicate statistically significant differences among the tested samples according to one-way ANOVA followed by Tukey’s HSD test (*p* < 0.05). IC_50_ represents the concentration required to inhibit 50% of enzyme activity under the specified assay conditions. Lower IC_50_ values indicate greater inhibitory potency. Kojic acid and acarbose were used as reference inhibitors for tyrosinase and α-amylase, respectively.

## Data Availability

The original data presented in this study are included in the article and Supplementary Materials. The underlying raw experimental data supporting the findings are available from the corresponding author upon reasonable request.

## Supplementary Materials

The following supporting information can be downloaded at https://www.mdpi.com/article/10.3390/ijms27209241/s1.

## References

[1] Yilmaz M.A., Cakir O., Zengin G., Izol E., Behcet L. (2024). The uprisal of a lost endemic edible species, *Micromeria cymuligera*: Comprehensive elucidation of its biological activities and phytochemical composition. Food Biosci..

[2] Mladenova T., Stoyanov P., Denev P., Dimitrova S., Katsarova M., Teneva D., Todorov K., Bivolarska A. (2021). Phytochemical Composition, Antioxidant and Antimicrobial Activity of the Balkan Endemic *Micromeria frivaldszkyana* (Degen) Velen. (Lamiaceae). Plants.

[3] Azab A. (2016). *Micromeria*: Chemistry And Medicinal Activities. Eur. Chem. Bull..

[4] Mohammadhosseini M., Venditti A., Flamini G., Sarker S., Kalaee M. (2022). The genus *Micromeria* Benth.: An overview on ethnobotany, chemotaxonomy and phytochemistry. Trends Phytochem. Res..

[5] Kremer D., Dunkić V., Radosavljević I., Bogunić F., Ivanova D., Ballian D., Stešević D., Matevski V., Ranđelović V., Eleftheriadou E. (2022). Phytochemicals and Their Correlation with Molecular Data in *Micromeria* and *Clinopodium* (Lamiaceae) Taxa. Plants.

[6] Alara O.R., Abdurahman N.H., Ukaegbu C.I. (2021). Extraction of phenolic compounds: A review. Curr. Res. Food Sci..

[7] Dai J., Mumper R.J. (2010). Plant Phenolics: Extraction, Analysis and Their Antioxidant and Anticancer Properties. Molecules.

[8] Dall’Acqua S., Sut S., Zengin G., Peron G., Elbasan F., Yildiztugay E., Bibi Sadeer N., Mahomoodally M.F. (2022). Phytochemical Screening, Antioxidant, and Enzyme Inhibitory Properties of Three *Prangos* Species (*P. heyniae*, *P. meliocarpoides* var. *meliocarpoides*, and *P. uechtritzii*) Depicted by Comprehensive LC-MS and Multivariate Data Analysis. Antioxidants.

[9] Ersoy E., Batal Yıldızhan R., Yılmaz M.A., Yeşil Y., Şahin H., Eroğlu Özkan E., Boğa M. (2024). Investigating the Pharmacological Potential of *Micromeria myrtifolia* Boiss. & Hohen.: Phenolic Profiling and Biological Activity Assessments. Commagene J. Biol..

[10] Abu-Gharbieh E., Ahmed N.G. (2016). Bioactive content, hepatoprotective and antioxidant activities of whole plant extract of *Micromeria fruticosa* (L) Druce ssp *serpyllifolia* F Lamiaceae against Carbon tetrachloride-induced hepatotoxicity in mice. Trop. J. Pharm. Res..

[11] Sharma R., Shachter A., Almog L., Oren G., Roynik-Toshner H., Dudai N. (2021). Genetic chemical variability of the volatiles and phenolic compounds in *Clinopodium serpyllifolium* subsp. *fruticosum* (L.) Bräuchler syn. *Micromeria fruticosa* (L.) Druce grown in Israel. Isr. J. Plant Sci..

[12] Sarikurkcu C., Andrade J.C., Ozer M.S., de Lima Silva J.M.F., Ceylan O., de Sousa E.O., Coutinho H.D.M. (2020). LC-MS/MS profiles and interrelationships between the enzyme inhibition activity, total phenolic content and antioxidant potential of *Micromeria nervosa* extracts. Food Chem..

[13] Sarikurkcu C., Hanine H., Sarikurkcu R.B., Sarikurkcu R.T., Amarowicz R. (2020). *Micromeria myrtifolia*: The influence of the extracting solvents on phenolic composition and biological activity. Ind. Crops Prod..

[14] Taskin T., Dogan M., Yilmaz B.N., Senkardes I. (2020). Phytochemical screening and evaluation of antioxidant, enzyme inhibition, anti-proliferative and calcium oxalate anti-crystallization activities of *Micromeria fruticosa* spp. *brachycalyx* and *Rhus coriaria*. Biocatal. Agric. Biotechnol..

[15] Tošić S.M., Ilić M., Svilar L., Nikolić J., Mitić M., Mitić V., Stankov Jovanović V.P. (2025). Conserved and Divergent Phytochemical Profiles in Native and Micropropagated *Micromeria croatica* (Pers.) Schott: An LC-HRMS Study Across Solvent Extracts. Plants.

[16] Brahmi F., Amri S., Bentaleb S., Elsebai F.M., Yalaoui-Guellal D., Madani K. (2019). Comparison of phenolic contents and biological potential of different polar extracts of *Micromeria graeca*, from Algeria. Curr. Nutr. Food Sci..

[17] Kefi S., Essid R., Papetti A., Abid G., Bouslama L., Aouani E., Tabbene O., Limam F. (2023). Antioxidant, antibacterial, and antileishmanial potential of *Micromeria nervosa* extracts and molecular mechanism of action of the bioactive compound. J. Appl. Microbiol..

[18] Duman H., Dirmenci T. (2017). A new species of *Micromeria* (Lamiaceae) from Koycegiz (Mugla, southwest of Turkey). Turk. J. Bot..

[19] Kargin Solmaz F.O., Sarikurkcu C., Istifli E.S., Tepe B. (2025). Phytochemical profiling and molecular docking analysis of *Micromeria aybalae* essential oil: In vitro and in silico evaluations. J. Essent. Oil Bear. Plants.

[20] Petersen M., Simmonds M.S. (2003). Rosmarinic acid. Phytochemistry.

[21] Singleton V.L., Orthofer R., Lamuela-Raventós R.M. (1999). Analysis of total phenols and other oxidation substrates and antioxidants by means of folin-ciocalteu reagent. Methods in Enzymology.

[22] Pękal A., Pyrzynska K. (2014). Evaluation of Aluminium Complexation Reaction for Flavonoid Content Assay. Food Anal. Methods.

[23] Beddiar H., Boudiba S., Benahmed M., Tamfu A.N., Ceylan Ö., Hanini K., Kucukaydin S., Elomri A., Bensouici C., Laouer H. (2021). Chemical Composition, Anti-Quorum Sensing, Enzyme Inhibitory, and Antioxidant Properties of Phenolic Extracts of *Clinopodium nepeta* L. Kuntze. Plants.

[24] Gopčević K., Grujić S., Arsenijević J., Džamić A., Veličković I., Izrael-Živković L., Medić A., Mudrić J., Soković M., Đurić A. (2022). Bioactivity and phenolics profile of aqueous and ethyl acetate extracts of *Satureja kitaibelii* Wierzb. ex Heuff. obtained by ultrasound-assisted extraction. Sci. Rep..

[25] Eruygur N., Ayaz F. (2021). Investigation of acetylcholinesterase, butyrylcholinesterase, α-glucosidase, α-amylase, and tyrosinase inhibition and antioxidant activity of methanol and water extracts from aerial parts of *Phlomis lycia* D. Don. Int. J. Phytocosmetics Nat. Ingred..

[26] Taskın T., Oksuz M., Rayaman E., Ermanoglu M., Taskın D., Acar A.G., Kılıç Ö., Çalışkan Salihi E., Yılmaz N.B., Elçioğlu H. (2025). Phytochemical analysis and in vitro biological activity assessment of extracts from *Micromeria myrtifolia*. Int. J. Environ. Health Res..

[27] Akhlaghipour I., Nasimi Shad A., Askari V.R., Maharati A., Baradaran Rahimi V. (2023). How caffeic acid and its derivatives combat diabetes and its complications: A systematic review. J. Funct. Foods.

[28] Ravaria P., Saxena P., Laksmi Bs S., Ranjan V., Abidi S.W.F., Saha P., Ramamoorthy S., Ahmad F., Rana S.S. (2023). Molecular mechanisms of neuroprotective offerings by rosmarinic acid against neurodegenerative and other CNS pathologies. Phytother. Res..

[29] Huang D., Ou B., Prior R.L. (2005). The Chemistry behind Antioxidant Capacity Assays. J. Agric. Food Chem..

[30] Prior R.L., Wu X., Schaich K. (2005). Standardized Methods for the Determination of Antioxidant Capacity and Phenolics in Foods and Dietary Supplements. J. Agric. Food Chem..

[31] Niki E. (2010). Assessment of Antioxidant Capacity in vitro and in vivo. Free Radic. Biol. Med..

[32] Nguyen V., Taine E.G., Meng D., Cui T., Tan W. (2024). Chlorogenic Acid: A Systematic Review on the Biological Functions, Mechanistic Actions, and Therapeutic Potentials. Nutrients.

[33] Saha R., Majie A., Baidya R., Sarkar B. (2024). Verbascoside: Comprehensive review of a phenylethanoid macromolecule and its journey from nature to bench. Inflammopharmacology.

[34] Odom J.D., Sutton V.R. (2021). Metabolomics in Clinical Practice: Improving Diagnosis and Informing Management. Clin. Chem..

[35] Kotha R.R., Tareq F.S., Yildiz E., Luthria D.L. (2022). Oxidative Stress and Antioxidants—A Critical Review on In Vitro Antioxidant Assays. Antioxidants.

[36] Sarikurkcu C., Karakus Z. (2026). Solvent-Dependent Phenolic Profiling and Bioactivities of *Medicago marina* L.: An Ultrasound-Assisted Extraction Approach. Molecules.

[37] Cittan M., Çelik A. (2018). Development and validation of an analytical methodology based on Liquid Chromatography–Electrospray Tandem Mass Spectrometry for the simultaneous determination of phenolic compounds in olive leaf extract. J. Chromatogr. Sci..

[38] Apak R., Güçlü K., Özyürek M., Esin Karademir S., Erçaǧ E. (2006). The cupric ion reducing antioxidant capacity and polyphenolic content of some herbal teas. Int. J. Food Sci. Nutr..

[39] Bahadori M., Sarikurkcu C., Yalcin O., Cengiz M., Gungor H. (2019). Metal concentration, phenolics profiling, and antioxidant activity of two wild edible *Melanoleuca* mushrooms (*M. cognata* and *M. stridula*). Microchem. J..

[40] Sarikurkcu C., Targan S., Ozer M.S., Tepe B. (2017). Fatty acid composition, enzyme inhibitory, and antioxidant activities of the ethanol extracts of selected wild edible plants consumed as vegetables in the Aegean region of Turkey. Int. J. Food Prop..

[41] Sarikurkcu C., Uren M.C., Kocak M.S., Cengiz M., Tepe B. (2016). Chemical composition, antioxidant, and enzyme inhibitory activities of the essential oils of three *Phlomis* species as well as their fatty acid compositions. Food Sci. Biotechnol..

[42] Sarikurkcu C., Uren M.C., Tepe B., Cengiz M., Kocak M.S. (2015). *Phlomis armeniaca*: Phenolic compounds, enzyme inhibitory and antioxidant activities. Ind. Crops Prod..

[43] Karakus Z., Sarikurkcu C. (2026). Organ-Specific Phenolic Profiles, Antioxidant Capacity, and Enzyme-Inhibitory Activities of the Turkish Endemic *Origanum bilgeri*. Pharmaceuticals.

[44] Hurlbert S.H. (1984). Pseudoreplication and the design of ecological field experiments. Ecol. Monogr..

[45] Lazic S.E. (2010). The problem of pseudoreplication in neuroscientific studies: Is it affecting your analysis?. BMC Neurosci..

[46] Sun T., Tanumihardjo S.A. (2007). An Integrated Approach to Evaluate Food Antioxidant Capacity. J. Food Sci..

