# Supplementary material for: Natural Phenolic Antioxidants from Micromeria aybalae: Solvent-Dependent Composition and Bioactivity
Source: Int J Mol Sci. 2026 Oct 18;27(20):9241. doi: 10.3390/ijms27209241 (PMC13705282; doi:10.3390/ijms27209241)
Supplement: Supplementary file 1 [file ijms-27-09241-s001.zip › ijms-4644362-supplementary.pdf]

Supplementary Materials

Section S.1 describes the analytical methods for phytochemical characterization, antioxidant measurements, and enzyme inhibitory assays. Supplementary Tables S1–S2 report LC–MS/MS analytical parameters and calibration characteristics; Tables S3–S4 present yield-adjusted phenolic contents and antioxidant assay equivalents relative to the initial dry plant material.

Section S.1: Analytical Methods for Phytochemical Characterization, Antioxidant Activity, and Enzyme Inhibitory Activity.

Phytochemical analysis

A simple, rapid, reproducible, and sensitive method, which was previously developed and validated, was used for the simultaneous determination of 31 phenolic compounds using LC–ESI–MS/MS. An Agilent Technologies 1260 Infinity liquid chromatography system hyphenated to a 6420 Triple Quad mass spectrometer was used for quantitative analyses. Chromatographic separation was carried out on a Poroshell 120 EC-C18 (100 mm × 4.6 mm I.D., 2.7 µm) column. The mobile phase configuration (0.1% formic acid/methanol) was selected based on the improved chromatographic resolution of isomeric compounds. The selected mobile-phase composition also provided higher sensitivity for several phenolic compounds. As a result, the mobile phase was made up from solvent A (0.1%, *v/v* formic acid solution) and solvent B (methanol). The gradient profile was set as follows: 0.00 min 2% B eluent, 3.00 min 2% B eluent, 6.00 min 25% B eluent, 10.00 min 50% B eluent, 14.00 min 95% B eluent, 17.00 min 95% B and 17.50 min 2% B eluent. The column temperature was maintained at 25°C. The flow rate was 0.4 mL min^{-1} and the injection volume was 2.0 µL. The tandem mass spectrometer was interfaced to the LC system via an ESI source. The electrospray source of the MS was operated in negative and positive multiple reaction monitoring (MRM) mode and the interface conditions were as follows: capillary voltage of −3.5 kV, gas temperature of 300°C and gas flow of 11 L min^{-1}. The nebulizer pressure was 40 psi. The analytes were identified by comparing their retention times and MRM ion transitions with those of authentic standards.

The analytical method was previously validated by Cittan and Çelik (2018), including assessments of calibration linearity, analytical recovery, and intra-day and inter-day precision. The published recovery values ranged from 78.1% to 108.7%, and the precision assessments supported the repeatability of the method. These parameters refer to the original method validation performed using an olive leaf matrix. Matrix-specific recovery and precision assessments were not independently established for the *M. aybalae* extracts in the present investigation. Chromatographic and mass spectrometric parameters and calibration characteristics are summarized in Supplementary Tables S1 and S2, respectively.

For each target analyte, identification was based on the agreement of chromatographic retention time and monitored multiple reaction monitoring (MRM) transitions with those of the corresponding authentic reference standard. Analytes yielding signals below their compound-specific limits of detection (LOD) were reported as not detected (ND). Accordingly, ND indicates that the analyte was not detected within the sensitivity limits of the applied LC–ESI–MS/MS method and should not be interpreted as definitive evidence of its chemical absence from the investigated extracts. The compound-specific LOD and limits of quantification (LOQ) are provided in Supplementary Table S2.

Supplementary Table S1. ESI–MS/MS Parameters and analytical characteristics for the Analysis of Target Analytes by MRM Negative and Positive Ionization Mode

Target compounds	Rt (min)	Precursor ion	MRM1 (CE, V)	MRM2 (CE, V)
Compounds analyzed by NI mode				
Gallic acid	8.891	168.9 [M − H]−	125.0 (10)	–
Protocatechuic acid	10.818	152.9 [M − H]−	108.9 (12)	–
3,4-Dihydroxyphenylacetic acid	11.224	167.0 [M − H]−	123.0 (2)	–
(+)-Catechin	11.369	289.0 [M − H]−	245.0 (6)	202.9 (12)
Pyrocatechol	11.506	109.0 [M − H]−	90.6 (18)	52.9 (16)
2,5-Dihydroxybenzoic acid	12.412	152.9 [M − H]−	109.0 (10)	–
4-Hydroxybenzoic acid	12.439	136.9 [M − H]−	93.1 (14)	–
Caffeic acid	12.841	179.0 [M − H]−	135.0 (12)	–
Vanillic acid	12.843	166.9 [M − H]−	151.8 (10)	122.6 (6)
Syringic acid	12.963	196.9 [M − H]−	181.9 (8)	152.8 (6)
3-Hydroxybenzoic acid	13.259	137.0 [M − H]−	93.0 (6)	–
Vanillin	13.397	151.0 [M − H]−	136.0 (10)	–
Verbascoside	13.589	623.0 [M − H]−	461.0 (26)	160.8 (36)
Taxifolin	13.909	303.0 [M − H]−	285.1 (2)	125.0 (14)
Sinapic acid	13.992	222.9 [M − H]−	207.9 (6)	163.8 (6)
p-Coumaric acid	14.022	162.9 [M − H]−	119.0 (12)	–
Ferulic acid	14.120	193.0 [M − H]−	177.8 (8)	134.0 (12)
Luteolin 7-glucoside	14.266	447.1 [M − H]−	285.0 (24)	–
Rosmarinic acid	14.600	359.0 [M − H]−	196.9 (10)	160.9 (10)
2-Hydroxycinnamic acid	15.031	162.9 [M − H]−	119.1 (10)	–
Pinoresinol	15.118	357.0 [M − H]−	151.0 (12)	135.7 (34)
Eriodictyol	15.247	287.0 [M − H]−	151.0 (4)	134.9 (22)
Quercetin	15.668	301.0 [M − H]−	178.6 (10)	151.0 (16)
Kaempferol	16.236	285.0 [M − H]−	242.8 (16)	229.1 (18)
Compounds analyzed by PI mode				
Chlorogenic acid	11.802	355.0 [M + H]+	163.0 (10)	–
(−)-Epicatechin	12.458	291.0 [M + H]+	139.1 (12)	122.9 (36)
Hesperidin	14.412	611.1 [M + H]+	449.2 (4)	303.0 (20)
Hyperoside	14.506	465.1 [M + H]+	303.1 (8)	–
Apigenin 7-glucoside	14.781	433.1 [M + H]+	271.0 (18)	–
Luteolin	15.923	287.0 [M + H]+	153.1 (34)	135.1 (36)
Apigenin	16.382	271.0 [M + H]+	153.0 (34)	119.1 (36)

R_t, retention time; NI, negative ion; and PI, positive ion.

Supplementary Table S2. Calibration curves and sensitivity properties of the method

Compounds	Linearity and sensitivity characteristics				
	Range (μg/L)	Linear equation	R^2	LOD (μg/L)	LOQ (μg/L)
Gallic acid	5–500	y = 4.82x − 26.48	0.9988	1.46	4.88
Protocatechuic acid	2.5–500	y = 5.65x − 9.99	0.9990	1.17	3.88
3,4-Dihydroxyphenylacetic acid	5–500	y = 5.13x − 12.39	0.9990	1.35	4.51
(+)-Catechin	10–500	y = 1.45x + 1.95	0.9974	3.96	13.20
Pyrocatechol	25–400	y = 0.11x − 0.52	0.9916	9.62	32.08
Chlorogenic acid	1–500	y = 12.14x + 32.34	0.9995	0.55	1.82
2,5-Dihydroxybenzoic acid	5–500	y = 3.79x − 14.12	0.9980	2.12	7.08
4-Hydroxybenzoic acid	5–500	y = 7.62x + 22.79	0.9996	1.72	5.72
(−)-Epicatechin	5–500	y = 9.11x − 9.99	0.9971	1.85	6.18
Caffeic acid	5–500	y = 11.09x + 16.73	0.9997	3.15	10.50
Vanillic acid	10–500	y = 0.49x − 1.61	0.9968	2.56	8.54
Syringic acid	10–500	y = 0.74x − 1.54	0.9975	3.75	12.50
3-Hydroxybenzoic acid	5–500	y = 3.69x − 12.29	0.9991	1.86	6.20
Vanillin	50–500	y = 2.02x + 135.49	0.9926	15.23	50.77
Verbascoside	2.5–500	y = 8.59x − 28.05	0.9988	0.82	2.75
Taxifolin	5–500	y = 12.32x + 9.98	0.9993	1.82	6.05
Sinapic acid	5–500	y = 2.09x − 6.79	0.9974	2.64	8.78
p-Coumaric acid	5–500	y = 17.51x + 53.73	0.9997	1.93	6.44
Ferulic acid	5–500	y = 3.32x − 4.30	0.9992	1.43	4.76
Luteolin 7-glucoside	1–500	y = 45.25x + 156.48	0.9996	0.45	1.51
Hesperidin	5–500	y = 5.98x + 0.42	0.9993	1.73	5.77
Hyperoside	2.5–500	y = 16.32x − 1.26	0.9998	0.99	3.31
Rosmarinic acid	1–500	y = 9.82x − 17.98	0.9989	0.57	1.89
Apigenin 7-glucoside	1–500	y = 21.33x − 31.69	0.9983	0.41	1.35
2-Hydroxycinnamic acid	1–500	y = 16.72x − 26.94	0.9996	0.61	2.03
Pinoresinol	10–500	y = 0.80x − 2.69	0.9966	3.94	13.12
Eriodictyol	2.5–500	y = 14.24x − 0.50	0.9998	0.80	2.68
Quercetin	5–500	y = 14.68x − 18.25	0.9997	1.23	4.10
Luteolin	5–500	y = 8.96x + 26.80	0.9992	1.34	4.46
Kaempferol	10–500	y = 0.82x − 3.06	0.9959	3.30	10.99
Apigenin	2.5–500	y = 11.29x + 38.05	0.9987	0.96	3.20

LOD and LOQ: limit of detection and limit of quantification, respectively.

Yield-adjusted contents relative to the initial dry plant material

The values in Supplementary Tables S3 and S4 were derived from the mean concentrations or standard-equivalent assay responses reported for the extracts in the main manuscript and the corresponding mean extraction yields (water: 11.14%; methanol: 2.45%; ethyl acetate: 1.45%). For each analyte or assay, the extract-based mean was multiplied by the extraction yield expressed as a decimal fraction (0.1114, 0.0245, or 0.0145). These calculations provide a complementary comparison on an initial dry plant material basis while retaining the original extract-based results in the main manuscript.

The mean total phenolic content (TPC) values were also adjusted for extraction yield to provide complementary estimates relative to the initial dry plant material. The resulting values were 7.98, 0.80, and 0.10 mg gallic acid equivalents (GAE)/g dry plant material for the water, methanol, and ethyl acetate extracts, respectively. These values represent calculated Folin–Ciocalteu assay equivalents and should not be interpreted as absolute recovery of all phenolic compounds.

Supplementary Table S3. Yield-adjusted phenolic contents of *M. aybalae* extracts expressed per gram of initial dry plant material (μg/g dry plant material).

Compound	Water	Methanol	Ethyl acetate
Gallic acid	0.55	0.55	ND
Protocatechuic acid	33.28	3.21	1.43
3,4-Dihydroxyphenylacetic acid	40.95	0.77	ND
(+)-Catechin	ND	0.65	ND
Pyrocatechol	ND	ND	ND
Chlorogenic acid	12.18	0.63	7.58
2,5-Dihydroxybenzoic acid	4.02	ND	ND
4-Hydroxybenzoic acid	3.79	1.21	ND
(−)-Epicatechin	ND	1.43	ND
Vanillic acid	8.31	2.50	ND
Caffeic acid	132.40	7.85	0.62
Syringic acid	19.24	2.42	ND
3-Hydroxybenzoic acid	ND	ND	ND
Vanillin	ND	1.16	ND
Verbascoside	20.55	3.82	18.68
Taxifolin	ND	0.25	ND
Sinapic acid	ND	ND	ND
p-Coumaric acid	1.04	0.17	ND
Ferulic acid	3.09	0.65	ND
Luteolin 7-glucoside	0.71	0.86	ND
Hesperidin	4.49	0.96	ND
Hyperoside	0.43	0.80	ND
Rosmarinic acid	2189.04	278.81	5.52
Apigenin 7-glucoside	4.87	3.98	ND
2-Hydroxycinnamic acid	ND	ND	ND
Pinoresinol	1.36	0.99	2.69
Eriodictyol	ND	ND	ND
Quercetin	ND	ND	ND
Luteolin	ND	ND	ND
Kaempferol	ND	ND	ND
Apigenin	ND	1.27	ND

Values are calculated from the mean analyte concentrations (μg/g extract; main manuscript, Table 2) multiplied by the corresponding mean extraction yields (main manuscript, Table 1). The estimates are reported to two decimal places, without SD or statistical comparison, because variability in extraction yields and analyte measurements was not propagated through these group-mean calculations. These are yield-adjusted content estimates, not independently determined absolute extraction recoveries. ND, not detected.

Supplementary Table S4. Yield-adjusted antioxidant assay equivalents of *M. aybalae* extracts expressed per gram of initial dry plant material.

Assay	Unit	Water	Methanol	Ethyl acetate
Phosphomolybdenum	mg TE/g dry plant	36.59	5.99	2.93
DPPH	mg TE/g dry plant	47.15	3.18	0.23
ABTS	mg TE/g dry plant	57.96	3.74	0.47
CUPRAC	mg TE/g dry plant	105.37	7.70	1.23
FRAP	mg TE/g dry plant	59.05	4.42	0.24
Metal chelating	mg EDTAE/g dry plant	2.51	0.28	0.38

Values were calculated by multiplying the mean assay equivalents per gram of extract (main manuscript, Table 3) by the corresponding mean extraction yield (main manuscript, Table 1). TE, Trolox equivalents; EDTAE, EDTA equivalents. Derived group-mean values are shown without SD or inferential statistical comparisons. These yield-adjusted standard-equivalent responses must not be interpreted as experimentally determined recoveries of specific antioxidant compounds or as absolute antioxidant recovery percentages.

Interpretation of yield-adjusted findings

After accounting for extraction yield, the aqueous protocol provided the highest calculated rosmarinic acid and caffeic acid contents per gram of initial dry plant material and the highest yield-adjusted responses in all six antioxidant assays. Although the ethyl acetate extract had the highest verbascoside concentration when expressed per gram of extract, its calculated verbascoside content per gram of starting dry plant was slightly lower than that of the aqueous extract. Likewise, the ethyl acetate extract exhibited the highest metal-chelating activity per gram of extract, whereas the aqueous protocol yielded the highest metal-chelating standard equivalents per gram of initial dry plant material. These descriptive comparisons reflect the combined effects of solvent, extraction conditions, and extraction yield; the present design does not isolate solvent polarity as the sole causal factor.

Biological activity

For total phenolic content, sample solution (0.25 mL) was mixed with diluted Folin-Ciocalteu reagent (1 mL, 1:9) and shaken vigorously. After 3 min, Na_2CO_3 solution (0.75 mL, 1%) was added and the sample absorbance was read at 760 nm after 2 h incubation at room temperature. Total phenolic content was expressed as equivalents of gallic acid.

For total flavonoid content, sample solution (1 mL) was mixed with the same volume of aluminium trichloride (2%) in methanol. Similarly, a blank was prepared by adding sample solution (1 mL) to methanol (1 mL) without $AlCl_3$. The sample and blank absorbance were read at 415 nm after 10 min incubation at room temperature. Absorbance of the blank was subtracted from that of the sample. Total flavonoid content was expressed as equivalents of rutin.

Total antioxidant activity of the samples was evaluated by phosphomolybdenum method. Sample solution (0.2 mL) was combined with 2 mL of reagent solution (0.6 M sulfuric acid, 28 mM sodium phosphate and 4 mM ammonium molybdate). The sample absorbance was read at 695 nm after 90 min incubation at 95°C.

For 1,1-diphenyl-2-picrylhydrazyl (DPPH) radical scavenging activity, sample solution (1 mL) was added to a 4 mL of 0.004% methanol solution of DPPH. Sample absorbance was read at 517 nm after 30 min incubation at room temperature in dark.

For ABTS cation radical scavenging activity, briefly, ABTS$^{\cdot+}$ radical cation was produced directly by reacting 7 mM ABTS solution with 2.45 mM potassium persulfate and allowing the mixture to stand for 12-16 h in dark at the room temperature. Prior to beginning the assay, ABTS solution was diluted with methanol to obtain an absorbance of 0.700 ± 0.02 at 734 nm. Sample solution (1 mL) was added to ABTS solution (2 mL) and mixed. Sample absorbance was read at 734 nm after 7 min incubation at room temperature.

For metal chelating activity on ferrous ions, briefly, sample solution (2 mL) was added to $FeCl_2$ solution (0.05 mL, 2 mM). The reaction was initiated by the addition of 5 mM ferrozine (0.2 mL). Similarly, a blank was prepared by adding sample solution (2 mL) to $FeCl_2$ solution (0.05 mL, 2 mM) and water (0.2 mL) without ferrozine. Then, the sample and blank absorbance were read at 562 nm after 10 min incubation at room temperature.

For cupric ion reducing activity (CUPRAC), sample solution (0.5 mL) was added to a premixed reaction mixture containing $CuCl_2$ (1 mL, 10 mM), neocuproine (1 mL, 7.5 mM) and NH_4Ac buffer (1 mL, 1 M, pH 7.0). Similarly, a blank was prepared by adding sample solution (0.5 mL) to a premixed reaction mixture (3 mL) without $CuCl_2$. Then, the sample and blank absorbance were read at 450 nm after 30 min incubation at room temperature.

For ferric reducing antioxidant power (FRAP), sample solution (0.1 mL) was added to a premixed FRAP reagent (2 mL) containing acetate buffer (0.3 M, pH 3.6), 2,4,6-tris(2-pyridyl)-s-triazine (TPTZ) (10 mM) in 40 mM HCl and ferric chloride (20 mM) in a ratio of 10:1:1 (v/v/v). Then, the sample absorbance was read at 593 nm after 30 min incubation at room temperature.

Inhibitory activity on α-amylase was performed using Caraway-Somogyi iodine/potassium iodide (IKI) method. Sample solution (25 µL) was mixed with α-amylase solution (50 µL) in phosphate buffer (pH 6.9 with 6 mM sodium chloride) in a 96-well micro plate and incubated for 10 min at 37°C. After pre-incubation, the reaction was initiated by the addition of starch solution (50 µL, 0.05%). Similarly, a blank was prepared by adding sample solution to all reaction reagents without enzyme solution (α-amylase). The reaction mixture was incubated 10 min at 37°C. The reaction was then stopped with the addition of HCl (25 µL, 1 M). This was followed by the addition of iodine-potassium iodide solution (100 µL). The sample and blank absorbance were read at 630 nm. Absorbance of the blank was subtracted from that of the sample.

Tyrosinase inhibitory activity was measured using a modified dopachrome method with L-DOPA as substrate. Sample solution (25 µL) was mixed with tyrosinase solution (40 µl) and phosphate buffer (100 µl, pH 6.8) in a 96-well microplate and incubated for 15 min at 25°C. The reaction was then initiated with the addition of L-DOPA (40 µl). Similarly, a blank was prepared by adding sample solution to all reaction reagents without enzyme (tyrosinase) solution. The sample and blank absorbance were read at 492 nm after 10 min incubation at 25°C.

The biological activities of the extracts were expressed as mg standard equivalent/g extract and compared with those of the standards, including trolox, ethylenediaminetetraacetic acid (disodium salt) (EDTA), kojic acid, and acarbose, used as positive controls.

Calculation of relative antioxidant capacity index (RACI)

The relative antioxidant capacity index (RACI) was calculated as a descriptive composite of standardized assay responses. For each included assay, extract values were standardized by subtracting the assay mean and dividing by the corresponding standard deviation. The RACI value for each extract

was then calculated as the arithmetic mean of its standardized values across the included measurements. This descriptive index was used to summarize assay-level variation among the extract types; it was not used to infer associations between individual phenolic compounds and biological activities.
